# Genetic basis of early onset and progression of type 2 diabetes in South Asians

**DOI:** 10.1038/s41591-024-03317-8

**Published:** 2024-11-26

**Authors:** Sam Hodgson, Alice Williamson, Margherita Bigossi, Daniel Stow, Benjamin M. Jacobs, Miriam Samuel, Joseph Gafton, Julia Zöllner, Marie Spreckley, Genes & Health Research Team, Sam Hodgson, Daniel Stow, Benjamin M. Jacobs, Miriam Samuel, Julia Zöllner, Marie Spreckley, Shaheen Akhtar, Ana Angel, Omar Asgar, Samina Ashraf, Saeed Bidi, Gerome Breen, James Broster, Raymond Chung, David Collier, Charles J. Curtis, Shabana Chaudhary, Grainne Colligan, Panos Deloukas, Ceri Durham, Faiza Durrani, Fabiola Eto, Joseph Gafton, Chris Griffiths, Joanne Harvey, Teng Heng, Qin Qin Huang, Karen A. Hunt, Matt Hurles, Shapna Hussain, Kamrul Islam, Vivek Iyer, Georgios Kalantzis, Ahsan Khan, Cath Lavery, Sang Hyuck Lee, Daniel MacArthur, Eamonn Maher, Daniel Malawsky, Sidra Malik, Hilary Martin, Dan Mason, Mohammed Bodrul Mazid, John McDermott, Caroline Morton, Bill Newman, Vladimir Ovchinnikov, Elizabeth Owor, Iaroslav Popov, Asma Qureshi, Mehru Raza, Jessry Russell, Stuart Rison, Nishat Safa, Annum Salman, Michael Simpson, John Solly, Michael Taylor, Richard C. Trembath, Karen Tricker, David A. Van Heel, Klaudia Walter, Jan Whalley, Caroline Winckley, Suzanne Wood, John Wright, Sabina Yasmin, Ishevanhu Zengeya, Claudia Langenberg, Rohini Mathur, Moneeza K. Siddiqui, Sarah Finer, Claudia Langenberg, David A. van Heel, Rohini Mathur, Moneeza K. Siddiqui, Sarah Finer

**Affiliations:** 1 Wolfson Institute of Population Health, Queen Mary University of London, London, UK; 2 Precision Healthcare University Research Institute, Queen Mary University of London, London, UK; 3 University College London, London, UK; 4 Blizard Institute, Queen Mary University of London, London, UK; 5 Bradford Teaching Hospitals NHS Foundation Trust, Bradford, UK; 6 Manchester University Hospitals, Manchester, UK; 7 King’s College London, London, UK; 8 Clinical Research Centre, Clinical Pharmacology and Precision Medicine, London, UK; 9 Social Action for Health (charity), London, UK; 10 William Harvey Research Institute, Queen Mary University of London, London, UK; 11 Manchester Genes & Health, Manchester, UK; 12 Wellcome Sanger Institute, Hinxton, UK; 13 Waltham Forest Council, London, UK; 14 Garvan Institute, Sydney, NSW Australia; 15 Aston University, Birmingham, UK; 16 University of Manchester, Manchester, UK; 17 School of Biological and Behavioural Sciences, Queen Mary University of London, London, UK; 18 Joint Research and Development Office, Queen Mary University of London, London, UK; 19 LCRN Greater Manchester Core Team, NIHR Clinical Research Network, Manchester, UK

**Keywords:** Genetics research, Type 2 diabetes, Disease genetics

## Abstract

South Asians develop type 2 diabetes (T2D) early in life and often with normal body mass index (BMI). However, reasons for this are poorly understood because genetic research is largely focused on European ancestry groups. We used recently derived multi-ancestry partitioned polygenic scores (pPSs) to elucidate underlying etiological pathways British Pakistani and British Bangladeshi individuals with T2D (*n* = 11,678) and gestational diabetes mellitus (GDM) (*n* = 1,965) in the Genes & Health study (*n* = 50,556). Beta cell 2 (insulin deficiency) and Lipodystrophy 1 (unfavorable fat distribution) pPSs were most strongly associated with T2D, GDM and younger age at T2D diagnosis. Individuals at high genetic risk of both insulin deficiency and lipodystrophy were diagnosed with T2D 8.2 years earlier with BMI 3 kg m^−^^2^ lower compared to those at low genetic risk. The insulin deficiency pPS was associated with poorer HbA1c response to SGLT2 inhibitors. Insulin deficiency and lipodystrophy pPSs were associated with faster progression to insulin dependence and microvascular complications. South Asians had a greater genetic burden from both of these pPSs than white Europeans in the UK Biobank. In conclusion, genetic predisposition to insulin deficiency and lipodystrophy in British Pakistani and British Bangladeshi individuals is associated with earlier onset of T2D, faster progression to complications, insulin dependence and poorer response to medication.

## Abstract (web summary)

In a cohort of 50,556 South Asian individuals, partitioned polygenic scores helped identify genetic susceptibility to insulin deficiency and unfavorable fat distribution as key drivers of young-onset T2D diagnosis and faster progression to diabetes-related complications.

## Main

Type 2 diabetes (T2D) is common, particularly in South Asian individuals, among whom the prevalence is estimated to be 12.7% globally and as high as 30% in Pakistan^1,2^. Compared to individuals of European ancestry, South Asians tend to be diagnosed with T2D at younger ages and with lower body mass index (BMI)^2,3^. This phenomenon exists even when the environment is not shared: Asian Indians are up to four times more likely to be diagnosed with T2D young (<40 years) and lean compared to Europeans^4^ and up to three times more likely to develop gestational diabetes mellitus (GDM)^5^.

South Asian individuals have historically been poorly represented in genetic studies, including those relating to diabetes^6,7^. This is pertinent because the recent characterization of T2D as a heterogeneous condition, comprising multiple subgroups or clusters (also referred to as endotypes), relies on phenotypic and genetic characterization to elucidate underlying etiology^8^. Phenotypic endotyping approaches have shown a preponderance of severe insulin-deficient diabetes (SIDD) in South Asians observed in two independent cohorts in India: 35% and 52%, respectively^9,10^. This contrasts with individuals of European ancestry, among whom insulin resistance plays a more prominent role^10^. Genetic endotyping has been largely restricted to individuals of European ancestry, with no representation of South Asian populations in more recent multi-ancestry efforts^11–14^. The latest multi-ancestry meta-genome-wide association study (GWAS) specifically highlights the utility of partitioned polygenic scores (pPSs) as a tool to characterize genetic endotypes and demonstrates their potential advantage over polygenic scores in identifying associations with diabetes-related complications. However, only 2% of the 2.5 million individuals in that study were of South Asian ancestry^15^.

Unlike polygenic scores that predict overall risk of an outcome (such as T2D) by summing the genetic burden of risk from genetic variants identified in GWASs, pPSs offer additional insight by constructing individual scores related to underlying etiological pathways. In recently published multi-ancestry pPS analyses that included participants of African, East Asian and European ancestry, this was achieved by leveraging high-throughput clustering approaches to identify genetic variants that clustered together and estimating 12 individual-level scores of the genetic burden of risk relating to underlying mechanistic pathways, such as beta cell dysfunction, obesity, lipid metabolism or lipodystrophy^16^. These have been shown to be associated with risk of diabetes complications—for example, a pPS for beta cell function, associated with insulin deficiency, was associated with impaired renal function^16,17^.

However, as many aspects of genetic architecture and clinical phenotype of T2D differ between South Asians and Europeans^9,18^, it remains unclear to what extent these pPSs might help to understand T2D risk and progression in individuals of South Asian ancestry, especially in comparison to global T2D polygenic risk scores (PRSs). Furthermore, the phenotypic characteristics associated with an individual having high genetic risk across multiple distinct etiological pathways, which we term ‘pPS extremes’, have not been explored but could offer opportunities to identify associations of genetically defined etiological endotypes with clinically relevant outcomes. We used Genes & Health, a long-term community-based study with genetics and linked electronic health and prescribing data for over 51,000 British Pakistani and British Bangladeshi individuals living in the United Kingdom (UK)^19^, to investigate whether pPS can help unravel the etiological factors driving young-onset T2D and clinically relevant related outcomes.

## Results

### Characteristics of populations studied

**Fig. 1:**
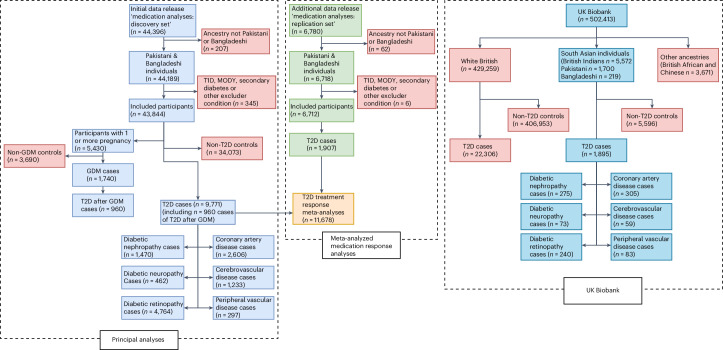
Included participants in the Genes & Health study and the UK Biobank. Participant flow diagram detailing the number of individuals enrolled in the Genes & Health study and included in each stage of analysis and the number of participants of similar ancestry enrolled in the UK Biobank.

We analyzed data from 9,771 British Pakistani and British Bangladeshi individuals with a T2D diagnosis and 34,073 diabetes-free controls (Fig. 1); demographic and clinical information stratified by ancestry and sex is shown in Table 1. Compared to Pakistani individuals, Bangladeshi individuals tended to be diagnosed with T2D earlier in life and at lower BMI and (females) had higher rates of prior GDM. Consequently, a higher prevalence of T2D was observed in Bangladeshis. However, at diagnosis and in the 5 years after diagnosis, Pakistanis had higher glycated hemoglobin (HbA1c) and BMI. Overall, Pakistanis were more likely to be prescribed insulin and develop nephropathy than Bangladeshis, despite being prescribed anti-diabetic medications from more classes.

**Table 1:** Demographic information stratified by genetically inferred ancestry

		All		s.e.	Bangladeshi	s.e.	Pakistani	s.e.	ANOVA P value
*n*		9,771			6,608		3,163		NA
T2D prevalence (%)		22.11		NA	23.36	NA	16.26	NA	6.50 × 10^−9^
Age at diagnosis (%)		46.65		0.12	45.18	0.14	50.03	0.21	1.56 × 10^−81^
HbA1c at diagnosis (mmol/mol)		60.1		0.21	59.61	0.26	60.88	0.43	0.00774825
HbA1c at diagnosis (in %)		7.63		0.026	7.61	0.033	7.67	0.054	0.00774825
HbA1c at 5 years from diagnosis (mmol/mol)		57.8		0.21	56.9	0.24	59.59	0.42	3.29 × 10^−9^
HbA1c at 5 years from diagnosis (in %)		7.45	0.0026		7.38	0.028	7.61	0.053	3.29 × 10^−9^
BMI at diagnosis – females (kg/m^2^)		31.03		0.11	30.06	0.12	33.2	0.22	2.74 × 10^−39^
BMI at diagnosis – male (kg/m^2^)		27.86		0.08	27.17	0.09	29.45	0.17	4.24 × 10^−39^
Nephropathy prevalence (%)		15.04		0.36	13.65	0.44	18.22	0.72	1.53 × 10^−8^
Neuropathy prevalence (%)		4.73		0.21	4.4	0.26	5.61	0.43	0.0124733
Diabetic eye disease prevalence (%)		48.76		0.51	48.78	0.63	48.76	0.93	0.986876
Insulin dependence prevalence (%)		23.35		0.43	22.81	0.53	24	0.8	0.21227
GDM prevalence (among females, %)		17.44		0.56	21.29	0.77	10.41	0.82	2.04 × 10^−18^
GDM to T2D prevalence (among females, %)		27.91		0.84	28.95	0.97	25.12	1.76	0.0813303
Medication classes prescribed in 5 years from diagnosis (*n*)		1.47		0.01	1.42	0.01	1.58	0.02	1.59 × 10^−11^

Comparisons between groups were performed using two-way ANOVA. HbA1c, glycated haemoglobin, presented in both mmol/mol and % units; s.e., standard error; NA, not applicable; T2D, type 2 diabetes; GDM, gestational diabetes melitus; GDM to T2D, incidence of type 2 diabetes after gestational diabetes mellitus.

### Ancestral differences in pPS distribution

**Extended Data Fig. 1:**
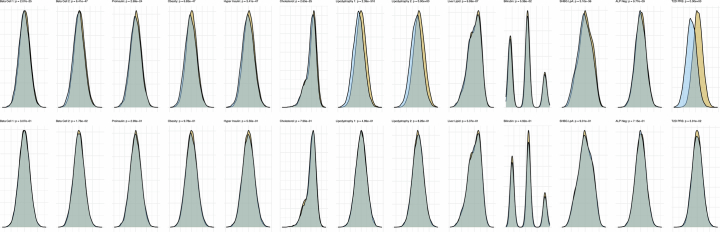
Partitioned polygenic score (pPS) distributions before and after ancestry adjustment. Density plots to show the distribution of 12 diabetes partitioned polygenic scores and one type 2 diabetes polygenic risk score before (top row) and after (bottom row) residual pPS/PRS calculation, by regressing out the effects of principal components 1–10 on each pPS/PRS, in 44,189 individuals in the genes & Health study. Plots are stratified by genetically-determined ancestry.

**Extended Data Fig. 2:**
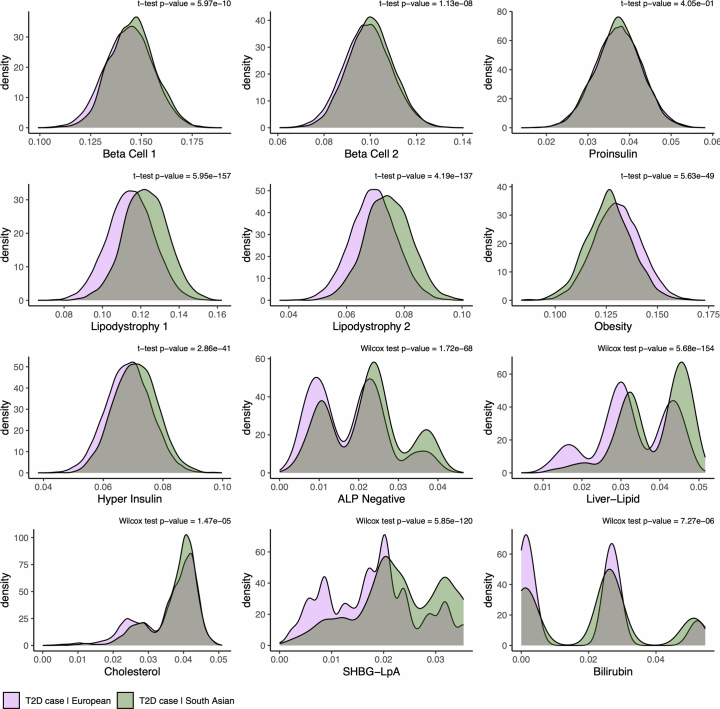
Comparison of pPS distributions between south Asians and white Europeans in UK biobank. Multi-ancestry pPS distribution comparisons. pPS distributions are shown for EUR and SAS individuals enrolled in UK Biobank. Test statistics are found in supplementary Table 4. The only non-significant difference was in the proinsulin pPS.

Noting the variation in clinical features of diabetes between British Bangladeshi and British Pakistani individuals in our cohort, we aimed to characterize the differences in genetic burden between them. To do this, we compared distributions of unmodified pPSs (not corrected for principal component (PC)-defined ancestry), observing higher unmodified pPSs in Bangladeshi individuals, notably for Beta Cell 2 (associated with lower HOMA-B with high proinsulin levels), Obesity (higher BMI) and Lipodystrophy 1(lower gluteofemoral fat and lower adiponectin levels) (Extended Data Fig. 1) (*P* value between Bangladeshis and Pakistanis <0.001 for all three pPSs). To maximize power in subsequent analyses, we pooled the two sub-ancestries using PC-corrected pPSs. We then compared distributions of pPSs between individuals with T2D of European and South Asian ancestry in the UK Biobank, observing higher scores among South Asians for several pPSs, including Beta Cell 2 (*P* = 3 × 10^−8^) and Lipodystrophy 1 (*P* = 1.95 × 10^−157^), whereas the Obesity pPS was higher among Europeans (*P* = 1.09 × 10^−47^) (Extended Data Fig. 2 and Supplementary Table 1).

### Association of pPSs and traits at time of diagnosis

**Extended Data Fig. 3:**
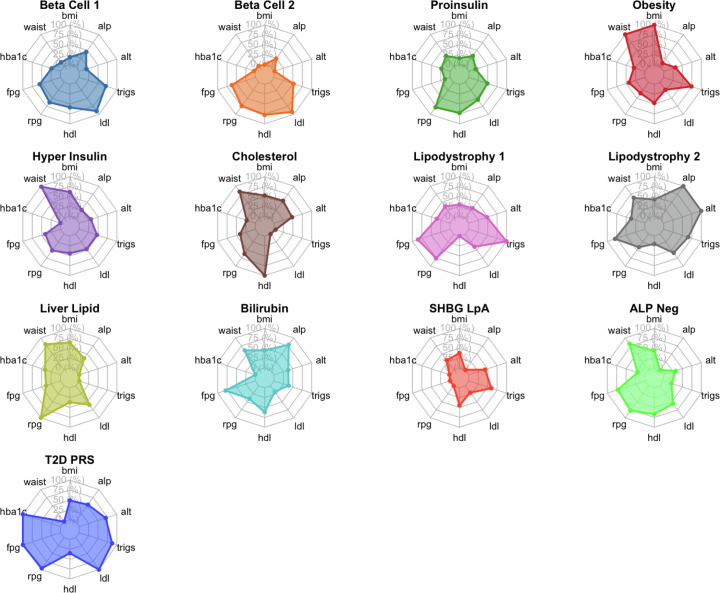
Association of pPS with metabolic traits at the time of type 2 diabetes diagnosis. Association between 12 diabetes partitioned polygenic scores (pPS) and z-normalised diabetes-related traits at the time of diagnosis in 9771 south Asian individuals with a diagnosis of type 2 diabetes. Data are presented as spider plots for each pPS, showing beta per standard deviation of pPS; the minimum value on each trait axis (comparable between pPS) represents the lowest beta for any pPS for that trait; the maximum value represents the highest beta for any pPS for that trait. EG, BMI and Waist circumference are highest in the ‘Obesity’ pPS; ALP is lowest in the ‘ALP Neg’ pPS. To allow comparison between traits, all traits were z-scored to a normal distribution with mean and median of 0 and standard deviation of 1. Only trait values within 1 year of diagnosis were included in analysis; where more than one trait was present, the value closest to time of diagnosis was used. ALP - alkaline phosphatase (n = 7422). ALT - alanine transferase (n = 7631). BMI - body mass index (n = 5791). HDL - high density lipoprotein (n = 7304). LDL = low density lipoprotein (n = 6365). RPG - random plasma glucose(n = 3777). FPG - fasting plasma glucose (n = 4786). Trigs - serum triglycerides (n = 6357). Waist - waist circumference (n = 1922). HbA1c = glycated haemoblogin (n = 7326).

**Extended Data Fig. 4:**
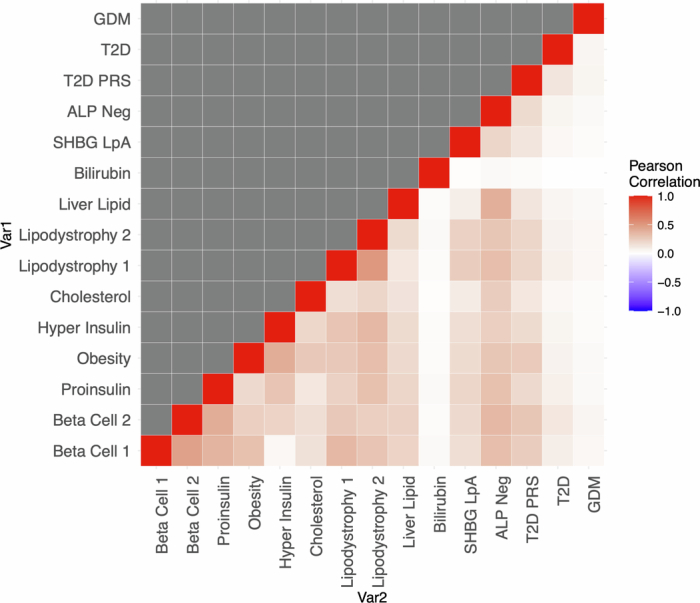
Correlation heatmap of pPS. Heatmap showing correlation between 12 diabetes partitioned polygenic scores, type 2 diabetes polygenic risk score (T2D PRS), type 2 diabetes status (T2D) and gestational diabetes mellitus status (GDM) in 43,844 individuals enrolled in the Genes & Health study.

We observed numerous expected associations between single pPSs and traits at time of diagnosis (Extended Data Fig. 3 and Supplementary Table 2) and correlations between pPSs (Extended Data Fig. 4 and Supplementary Table 3); further details are provided in the Supplementary Information.

### pPSs are associated with T2D, GDM and incident T2D after GDM

**Fig. 2:**
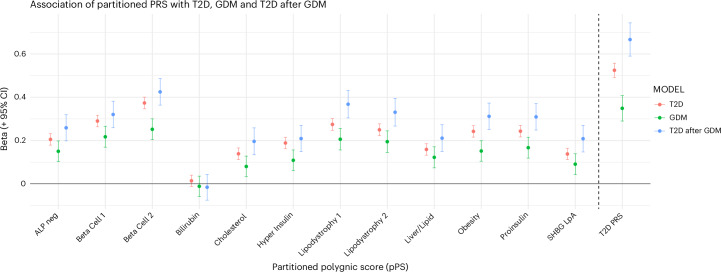
Association of pPSs with T2D and GDM risk. Association of pPSs with incident T2D (*n* = 9771), GDM (*n* = 1740) and T2D after GDM (*n* = 960) in 43,844 individuals in the Genes & Health study. Results for each pPS are presented as beta per s.d. of pPS with 95% CIs after adjustment for sex, age and ancestry. All associations remained statistically significant after Bonferroni correction (other than Bilirubin pPS, which was not associated with any outcome) (Supplementary Table 4). neg, negative.

**Extended Data Fig. 5:**
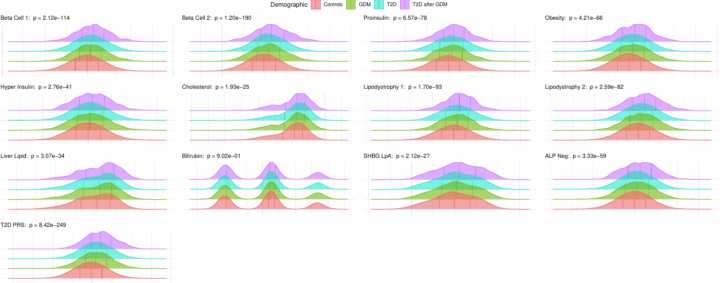
Stratified distributions of pPS by type 2 diabetes and gestational diabetes status. Distribution of 12 ancestry-corrected partitioned polygenic scores, and type 2 diabetes polygenic risk score (T2D PRS), in 43,844 individuals enrolled in Genes & Health. Density plots are presented for each score, stratified by T2D status (non-diabetic control; gestational diabetes mellitus (GDM); type 2 diabetes (T2D); and individuals developing T2D after GDM. p values for each plot compare differences between strata – two-way ANOVA was used for normally-distributed variables, and Kruskal Wallis test for non-normal variables (bilirubin).

As would be expected from the greater number of contributing single-nucleotide polymorphisms (SNPs) and the inclusion of SNPs from distinct mechanistic pathways, and in keeping with previously reported multi-ancestry results^16^, the global T2D PRS was more strongly associated with T2D, GDM and incident T2D after GDM than any individual pPS (Fig. 2) (T2D: beta per s.d. 0.52, 95% confidence interval (CI): 0.49–0.56; GDM: beta per s.d. 0.35, 95% CI: 0.29–0.40; T2D after GDM beta per s.d. 0.66, 95% CI: 0.59–0.74). For all pPSs other than Bilirubin, scores were higher among T2D cases, GDM cases and individuals with incident T2D after GDM, compared to non-diabetic controls (Extended Data Fig. 5). We observed associations between pPS and T2D risk, after adjustment for sex and ancestry, in 43,844 individuals (number of T2D cases = 9,771) (Fig. 2 and Supplementary Table 4). As in previous multi-ancestry analyses^16^, the strongest associations between pPS and T2D were observed for beta cell function-mediated endotypes: Beta Cell 1—related to glucose sensing (beta per s.d. 0.39, 95% CI: 0.35–0.42, *P* < 0.001); and Beta Cell 2—related to insulin deficiency (beta per s.d. 0.32, 95% CI: 0.28–0.35, *P* < 0.001); all pPS and T2D PRS associations remained statistically significant after Bonferroni correction.

After Bonferroni correction, all pPSs except Bilirubin were associated with GDM and incident T2D after GDM in 5,430 individuals with a history of at least one pregnancy (Fig. 2 and Supplementary Table 4). The strongest association between pPS and GDM was observed with Beta Cell 1 (beta per s.d. 0.24, 95% CI: 0.18–0.29) and Beta Cell 2 (beta per s.d. 0.23, 95% CI: 0.17–0.28) pPSs. Similar associations were observed between pPS and incident T2D after GDM, with strongest associations for Beta Cell 1 and Beta Cell 2.

### pPS and earlier age of diagnosis

**Fig. 3:**
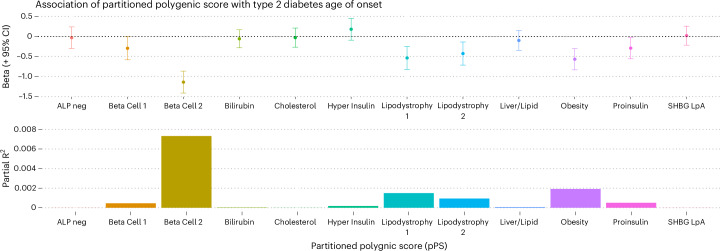
Association of pPS with T2D age of onset in 9,771 British Pakistani and British Bangladeshi individuals in the Genes & Health study. **a**, Association between 12 T2D pPSs and age at diagnosis of T2D, presented as beta (in years) per s.d. of pPS, estimated from a multivariable logistic regression model incorporating all 12 pPSs and adjusted for sex and ancestry. **b**, Partial R^2^ for effect of 12 diabetes pPSs on age at T2D diagnosis, estimated from the same model. After correction for multiple testing, only Beta Cell 2, Lipodystrophy 1 and Obesity pPSs were associated with age at diagnosis. neg, negative.

**Extended Data Fig. 6:**
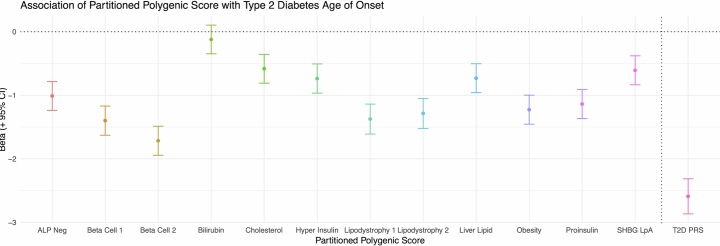
Unadjusted association of pPS with age at type 2 diabetes diagnosis. Association of twelve partitioned polygenic scores and a global type 2 diabetes polygenic risk score (T2D PRS) with type 2 diabetes age of onset in 9771 individuals. Results are presented as beta per standard deviation in score. Each beta was estimated from a separate multivariable regression model, adjusted for sex and ancestry.

The association between pPS and age of T2D diagnosis has not, to our knowledge, been explored before for any ancestry group. All pPSs other than Bilirubin were associated with earlier age of T2D onset, as was the T2D PRS, which showed the strongest association (Extended Data Fig. 6). However, many of the pPS effects were non-significant in a multivariable regression model incorporating all 12 pPSs (Fig. 2a). The pPSs associated with age of diagnosis after Bonferroni correction were Beta Cell 2 (diagnosis 1.1 years earlier in life per s.d., 95% CI: 0.9–1.4 years, *P* = 3 × 10^–16^), Obesity (0.57 years per s.d., 95% CI: 0.30–0.83 years, *P* = 3 × 10^–5^) and Lipodystrophy 1 (0.54 years per s.d., 95% CI: 0.25–0.82 years, *P* = 2 × 10^–4^), whereas other pPSs were nominally associated (Fig. 3a, Extended Data Fig. 6 and Supplementary Table 5). In this model, the pPS with the highest partial R^2^ was Beta Cell 2 (0.007), followed by Obesity (R^2^ = 0.002) and Lipodystrophy 1 (R^2^ = 0.001) (Fig. 3b).

**Extended Data Fig. 7:**
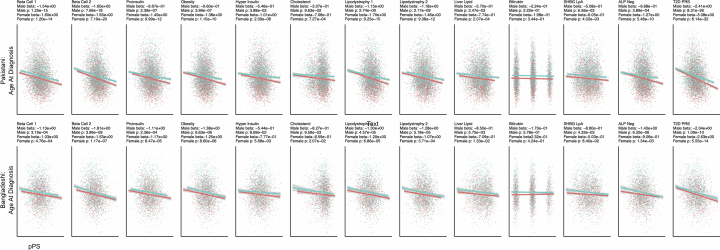
Association of pPS with age at type 2 diabetes diagnosis, stratified by sex and ancestry. Sex- and ancestry-stratified associations between 12 partitioned polygenic scores (pPS), a type 2 diabetes polygenic risk score (T2D PRS), and age at diagnosis of type 2 diabetes, in 9771 male (Blue line) and female (red line) Pakistani (bottom row) and Bangladeshi (top row) individuals with type 2 diabetes in the Genes & Health study. Sex- and ancestry-specific lines of best fit with 95% confidence intervals are plotted for each pPS, with male and female betas estimated from univariate logistic regression models regressing age at diagnosis on each pPS presented in each panel heading.

In ancestry-stratified and sex-stratified analyses, we found strongest effects for both Beta Cell 2 and Lipodystrophy 1 in Bangladeshi females (beta per s.d. = 1.93, *P* = 7.2 × 10^–20^ and beta per s.d. = 1.70, *P* = 9.3 × 10^–15^, respectively) (Extended Data Fig. 7). We found no significant interaction between pPSs and sex in ancestry-specific analyses in individuals of either Pakistani or Bangladeshi ancestry (Extended Data Fig. 7). Subsequent analyses were focused on Beta Cell 2, Obesity and Lipodystrophy pPSs as key drivers of early-onset T2D risk.

### pPS and response to diabetes-controlling medication

**Fig. 4:**
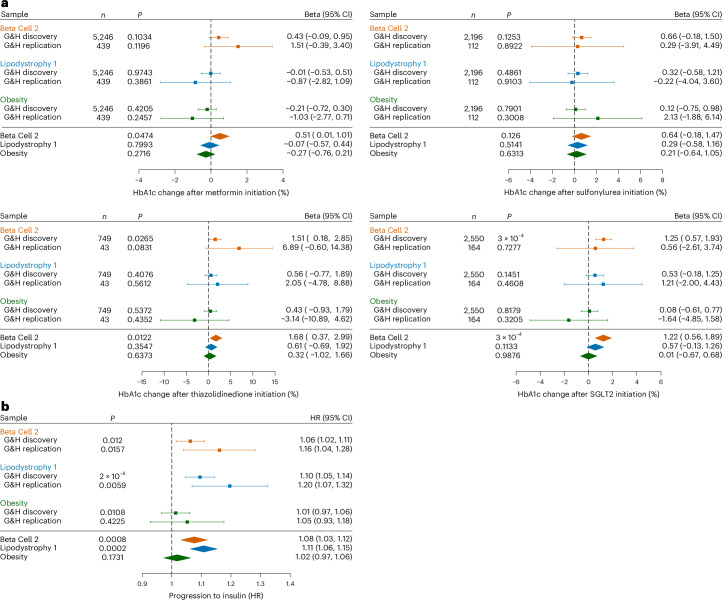
Association of pPSs with anti-diabetic medication initiation and response. **a**, Association of pPSs with change in HbA1c in response to medication initiation, presented as beta per s.d. (± 95% CIs and two-sided *P* values from *t* statistic), estimated from multivariable regression models adjusted for sex and ancestry. The change presented is mean percent change in HbA1c from pre-treatment to on-treatment; HbA1c units are mmol mol^−1^. After adjustment for multiple testing, the only association that was statistically significant was that of Beta Cell 2 pPS with SGLT2i response (further details are presented in Supplementary Table 6). Sulfonylurea, insulin secretagogues, including sulfonylureas and meglitinides (*n* = 2,196); Metformin, metformin (*n* = 5,246); SGLT2i, sodium/glucose co-transporter 2 inhibitors (*n* = 2,550); Thiazolidinedione, pioglitazone/thiazolidinediones (*n* = 749). **b**, Insulin-free survival from time of T2D diagnosis in 9,756 individuals for whom prescribing data were available (number of cases = 1,756), presented as HRs (± 95% CIs) estimated from Cox proportional hazard survival models adjusted for sex and genetically determined ancestry; presented *P* values are two-sided. Results for Beta Cell 2 and Lipodystrophy 1 pPSs were statistically significant after adjustment for multiple testing (Supplementary Table 7). G&H, Genes & Health.

**Extended Data Fig. 8:**
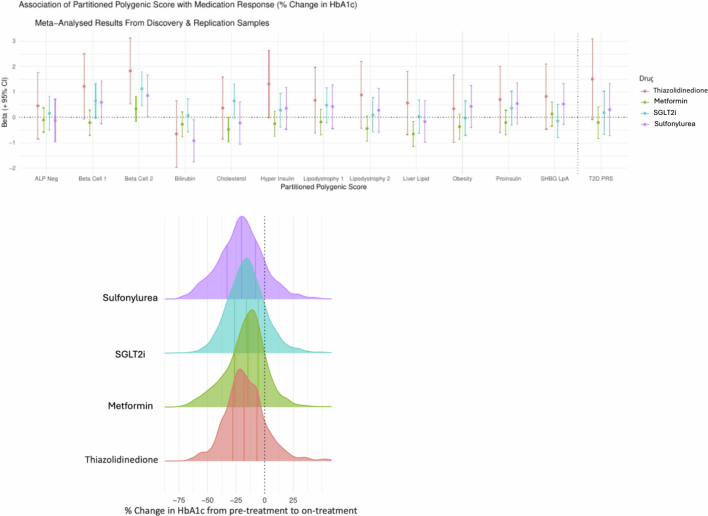
Partitioned polygenic scores and response to diabetes-controlling medication. Top panel: Association of pPS with mean change in HBA1c (+/− 95% confidence intervals) in response to medication initiation, presented as beta per standard deviation, estimated from multivariable regression models adjusted for sex and ancestry. Bottom panel: Distribution of percentagechange in HbA1c after initiation of 5 classes of oral antidiabetic medication, calculated as on-treatment HbA1c minus pre-treatment HbA1c, expressed as a percentage of pre-treatment HbA1c. Sulfonylurea = insulin secretagogiues (sulfonylureas and meglitinides, n = 2196), Metformin = metformin (n = 5246), SGLT2i = sodium/glucose contransporter 2 inhibitors (n = 2550), Thiazolidinedione = pioglitazone / thiazolidinediones (n = 749).

The association between pPS and response to medication has not, to our knowledge, been explored previously in any ancestry group. We observed associations between pPS and response to oral anti-diabetic medications, measured as the percent change in HbA1c (in mmol mol^−1^) after medication initiation (Fig. 4a, Extended Data Fig. 8 and Supplementary Table 6). We replicated our initial findings in a replication sample of 6,712 individuals (number of T2D cases = 1,907) in Genes & Health and then meta-analyzed our discovery and replication results. Higher scores across all pPSs were generally associated with negative, or no, response to oral anti-diabetic medication initiation (Extended Data Fig. 8a), whereas. across the whole sample of included participants, HbA1c typically declined after initiation of each medication class (median percent change in HbA1c ranged from −14.6% (metformin) to −20.3% (sulfolnylurea)) (Extended Data Fig. 8b). HbA1c values before initiation of metformin, sulphonyureas, sodium/glucose co-transporter 2 inhibitor (SGLT2i) and thiazolidinediones were 63.5, 77.2, 76.1 and 76.3 mmol mol^−1^, respectively. Higher Beta Cell 2 pPS score was associated with increased HbA1c after initiation of thiazolidinediones (meta-analyzed HbA1c increase 1.68% per s.d., 95% CI: 0.37–2.99, *P* = 0.01), SGLT2i (1.22% per s.d., 95% CI: 0.56–1.89, *P* = 3 × 10^−4^) and metformin (0.51%, 95% CI: 0.01–1.01, *P* = 0.047), although only the association with SGLT2i remained after Bonferroni correction (Supplementary Table 6). Higher Liver/Lipid pPS was nominally associated with decrease in HbA1c after initiation of metformin (−0.65% per s.d., 95% CI: −0.16 to 1.13, *P* = 0.008, did not pass correction for multiple testing). Interestingly, the T2D PRS was not associated with treatment response to metformin, thiazolidinediones, SGLT2 inhibitors or sulfonylureas (Extended Data Fig. 8a) either before or after Bonferroni correction. These findings highlight the etiological specificity of pPS relative to PRS.

### pPS and progression to insulin treatment

**Extended Data Fig. 9:**
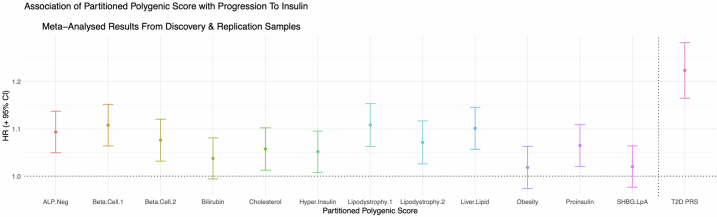
Partitioned polygenic scores and progression to insulin. Association between twelve diabetes partitioned polygenic scores (pPS) and a global type 2 diabetes polygenic risk score (T2D PRS) and progression from time of type 2 diagnosis to insulin therapy initiation, meta-analysed from discovery and replication samples, in 11,678 individuals followed up for 138,769 person-years post-diagnosis. Data are presented as Hazard ratios (HR) +/− 95% confidence intervals ascertained from cox proportional hazards survival models adjusted for sex and genetically determined ancestry.

**Extended Data Fig. 10:**
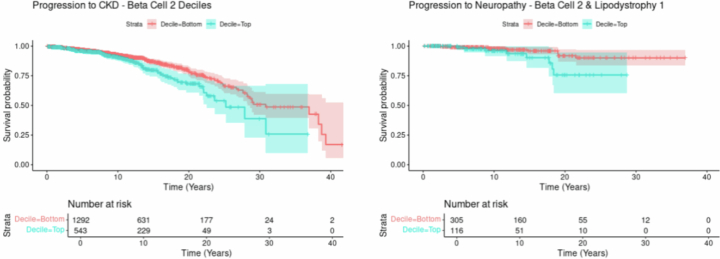
Extremes of genetic risk and progression to complications. Kaplan-Meier plots for progression to complications (nephropathy and neuropathy) from time of type 2 diabetes diagnosis in 9771 individuals in Genes & health. Plots are presented for only those results which were statistically significant in multivariable survival models adjusted for age and ancestry (Fig. 4c). Strata are defined as ‘extremes of genetic risk’ - in the left-hand plot, individuals in the top decile of the Beta Cell 2 pPS distribution are compared to those in the bottom decile. In the right hand plot, individuals in the top deciles of both the beta Cell 2 and Lipodystrophy 1 distributions are compared to those in the bottom of both distributions. Shaded areas indicate 95% confidence intervals.

The association between pPS and rate of progression to insulin treatment has not, to our knowledge, been explored previously in any ancestry group. Faster progression to insulin treatment is associated with earlier onset and poorly controlled T2D^20^. Therefore, the T2D PRS was associated with progression to insulin (hazard ratio (HR) per s.d. 1.22, 95% CI: 1.16–1.28) (Extended Data Fig. 10). The Beta Cell 2 and Lipodystrophy 1 pPSs were associated with progression to insulin treatment in Bonferroni-corrected adjusted survival models (HR per s.d. 1.08, 95% CI: 1.03–1.12, *P* = 0.0008; HR per s.d. 1.11 95% CI: 1.06–1.15, *P* = 0.0002), whereas the Obesity pPS was not associated with progression to insulin treatment irrespective of adjustment for multiple testing (Fig. 4b, Extended Data Fig. 9 and Supplementary Table 7).

### Extreme genetic risk, T2D phenotype and complications

**Fig. 5:**
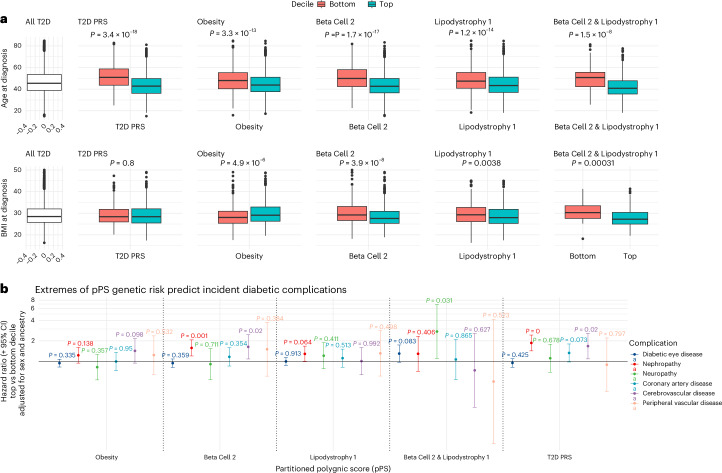
pPS genetic risk extremes, T2D phenotype and complications. Extremes of genetic risk association with age (**a**) and BMI (**b**) at diagnosis and progression to microvascular complications (**c**), among 9,771 individuals with T2D in the Genes & Health study. For **a** and **b**, box plots are presented contrasting individuals in the top and bottom 10% of the genetic risk distributions for three key pPSs (Obesity (*n* = 1,120 top decile/708 bottom decile), Beta Cell 2 (*n* = 1,309 top/566 bottom) and Lipodystrophy 1 (*n* = 1,164 top/635 bottom)) and a global T2D PRS (*n* = 1,385 top/508 bottom) and for individuals in the top and bottom 10% of both the Beta Cell 2 and Lipodystrophy distributions (*n* = 291 top/83 bottom) (right-most panel). Distributions for all individuals with T2D are presented in the left-most panel for comparison (*n* = 9,771). The middle line of each box represents the median value; the upper and lower bounds of the box represent the upper and lower quartiles; and the whiskers are defined as upper or lower quartile plus or minus 1.5 times the interquartile range. Distributions were compared using two-way ANOVA; all statistically significant associations remained after Bonferroni correction. For **c**, HRs are presented for each genetic risk extreme comparison, comparing complication-free survival from diagnosis between the bottom 10% of each pPS distribution (reference) and the top 10%. HRs were estimated from Cox proportional hazard models adjusted for sex and ancestry. After Bonferroni correction, only associations between nephropathy and Beta Cell 2 and T2D PRS remained significant (Supplementary Table 8). Further data are presented in Supplementary Table 9 (Schoenfeld residuals for survival models) and Extended Data Fig. 10 (illustrative Kaplan–Meier survival plots for positive results).

Finally, we explored whether extremes of genetic risk, defined as being in the top versus bottom decile of a pPS distribution (high or low risk, respectively), were associated with T2D clinical features (Supplementary Table 8). Across all participants, T2D prevalence was 22.1% with mean age of onset of 46.6 years. Among individuals in the top decile of the Lipodystrophy 1 distribution, prevalence of T2D was 26.3% (mean age of onset 44.6 years) (Fig. 5a). Among individuals in the top 10% of the Beta Cell 2 distribution, T2D prevalence was 29.6%, and mean age of onset was 43.6 years. Compared to individuals in the bottom 10% of the Beta Cell 2 pPS distribution, these individuals were more likely to develop nephropathy (HR 1.58, 95% CI: 1.19–2.06, *P* = 0.001, significant after correction for multiple testing) (Fig. 5c, Extended Data Fig. 10 and Supplementary Table 9). The Obesity pPS, in contrast, was not associated with progression to complications irrespective of Bonferroni correction.

The association of combined extremes of pPS with features such as age and BMI at diagnosis have not, to our knowledge, been explored previously. We observed that individuals at combined high genetic risk for Lipodystrophy 1 and Beta Cell 2 (in the top decile of both distributions, *n* = 110) were, on average, diagnosed with diabetes 8 years earlier in life than those in the bottom decile of both distributions (*n* = 304); had 3 kg m^−^^2^ lower BMI at diagnosis (Fig. 5a,b); had 7.8% higher lifetime prevalence of diabetic retinopathy; had 12% higher prevalence of insulin dependence; and had 3.73 mmol mol^−1^ higher 5-year HbA1c despite similar baseline HbA1c (Supplementary Table 8). In survival models, we found that they were nominally more likely to progress to diabetic neuropathy (HR 2.75, 95% CI: 1.10–6.9, *P* = 0.031), although this did not pass Bonferroni correction (Fig. 4c, Extended Data Fig. 10 and Supplementary Tables 9 and 10).

In Fig. 5, we show the association of pPS and T2D PRS with the same clinically relevant outcomes (further details in the Supplementary Information and Supplementary Table 10). The pPS–complication associations are broadly in keeping with a previously reported multi-ancestry analysis^16^, but we demonstrate, to our knowledge for the first time, the ability of pPS compared to PRS in identifying leaner onset T2D.

## Discussion

In this study of 50,556 British Pakistani and British Bangladeshi individuals, we show that multi-ancestry T2D pPSs developed in European, East Asian and African ancestry individuals are applicable to British South Asian individuals, where they are similarly associated with both T2D and GDM. We identified genetic susceptibility to insulin deficiency and unfavorable fat distribution as key drivers of T2D diagnosis at a younger age, at lower BMI and faster progression to diabetes-related complications and insulin dependence—individuals at high risk of both are diagnosed 8 years earlier in life at 3 kg m^−^^2^ lower BMI. Furthermore, we show that the Beta Cell 2 pPS, predictive of insulin deficiency, is associated with poorer response to commonly prescribed oral anti-diabetic medications and that South Asian individuals have greater genetic risk than European ancestry individuals. These results highlight the heterogeneity of T2D and specific etiologies underlying T2D risk in South Asians as well as a greater genetic predisposition to insulin deficiency and unfavorable fat distribution compared to Europeans.

Our results highlight the need to consider the etiological heterogeneity of T2D in clinical care pathways and indicate that additional clinical phenotyping may add benefit to the precision treatment of South Asian individuals with T2D—for example, measurement of C-peptide to estimate beta cell function. However, it is unclear whether the deployment of pPSs themselves in a clinical setting will add value.

Using the UK Biobank, we observed lower genetic predisposition to obesity in South Asian than European individuals but greater burden of Beta Cell 2 and Lipodystrophy 1 pPSs, both of which were associated with early and lean onset T2D (Fig. 5a,b and Extended Data Fig. 5). In Genes & Health, using stratified analyses, we identified higher unmodified (not corrected for PCs) genetic risk of all pPSs in Bangladeshi compared to Pakistani individuals (Extended Data Fig. 1). Although we were underpowered to observe interactions between ancestry and sex, we observed that Bangladeshi females showed the strongest associations between genetic risk and earlier onset T2D. These findings argue to an extent against the pooling of these distinct ancestral groups under the banner of ‘South Asian/SAS’ in genetic epidemiological studies when eventually sample size is no longer a limitation^21^. These findings also highlight important sex-stratified effects that can explain higher observed risk of T2D and GDM in females of certain ancestries. These findings sit alongside multi-ancestry comparisons made previously, most recently by Smith et al.^16^ who observed greater risk of Beta Cell 2 and Lipodystrophy 1 in East Asians relative to Europeans, and comparisons of previous beta cell deficiency pPSs showed a greater genetic burden in Asian Indians compared to Europeans^4^.

We identified genetic propensity to impaired insulin secretion as a key driver of the genetic basis of age at diagnosis of T2D in British Pakistanis and British Bangladeshis (Figs. 3 and 5a). This finding supports previous epidemiological observations of South Asians having lower HOMA-B (an estimate of beta cell function), lower BMI and greater dyslipidemia at the time of diagnosis as compared to Europeans^22^. The observed effect of greater genetic burden of impaired insulin secretion builds on previous findings from Asian Indians^4^, replicating this prior association with a newer multi-ancestry pPS and extending it to highlight specific etiologies underlying GDM as well as T2D treatment response, progression to insulin dependence and nephropathy.

Our observation that a genetic predisposition to unfavorable fat distribution plays a role in early onset and rapid progression of T2D in South Asians supports the well-recognized clinical phenotype of T2D at lower BMI and greater waist circumference compared to Europeans^23^.

There are limited studies that explore the underlying architecture of age of diagnosis and, by proxy, earlier onset of T2D^18,24^. However, efforts to do so agree with our findings that drivers of earlier age of diabetes onset do not overlap entirely with overall drivers of T2D risk. Our use of pPSs aids the identification of specific etiologies driving earlier onset in South Asians.

We observed an effect of Obesity pPS in driving early-onset T2D; however, the pPS was not associated with greater risk of insulin dependence or diabetes-related complications. This effect is mirrored in epidemiological studies that showed that South Asians who develop T2D at younger ages have increased weight over those who do not but that this weight difference is relatively smaller than that observed in Europeans or Black individuals^3^. This was also observed in a national survey of Asian Indians, where 45% of young diagnosed (<40 years) T2D was in individuals with obese (45%) or overweight (15%) BMI^25^. This study used genetics to uncover the role of these two adiposity-related etiologies (unfavorable fat distribution and BMI) in South Asian T2D.

In the present study, we show that the genetic architecture of GDM, assessed by association with genetically determined T2D endotypes, closely resembles that of the genetic basis of T2D itself in British Bangladeshis and British Pakistanis (Fig. 2 and Extended Data Fig. 5), in keeping with recent studies in European ancestry individuals^26^. In our study, the strongest associations with GDM were observed for Beta Cell 1 and Beta Cell 2, suggesting that genetic predisposition to insulin deficiency in British South Asian females may contribute to GDM risk as well as T2D risk. The association of GDM with the Obesity pPS in our study, in contrast, was relatively weaker, despite this being highlighted as a major etiological pathway in European^26^ ancestry and Turkish^27^ mothers with GDM.

We identified individuals at extremes of genetic risk (defined by pPS) who are at particularly high risk of developing T2D in early adulthood (Fig. 5a), responding poorly to widely used oral anti-diabetic drugs (Fig. 4a) and progressing rapidly to insulin requirement (Fig. 4b) and complications (Fig. 5c). Using pPSs to characterize genetic T2D endotypes could have additional utility above phenotypic characterization at disease onset given that they can be determined and are stable at any point in the life course.

Due to their etiological specificity, pPSs were associated with response to medication, whereas the T2D PRS was not. Overall, most individuals responded to the introduction of glucose-lowering medication with a reduction in HbA1c (Extended Data Fig. 8a). However, high genetic risk of insulin deficiency determined by high Beta Cell 2 pPS was associated with increased HbA1c after initiation of metformin, SGLT2 inhibitors and thiazolidinediones (Fig. 5a). These findings are biologically plausible considering that the mechanism of action of these drugs does not lead to greater insulin secretion—for example, the mode of action of thiazolidinediones as insulin sensitizers^28^ means that they are unlikely to benefit individuals if insulin deficiency is underpinning hyperglycemia. In contrast, we did not observe differential treatment response using a T2D polygenic score, highlighting the value of pPSs in dissecting clinically relevant pathophysiologies. Furthermore, our finding that T2D PRS is not associated with phenotypic traits, such as BMI (Fig. 5b), highlights its inability to elucidate specific etiologies. Our observed association between combined high genetic risk of Beta Cell 2 and Lipodystrophy 1 and neuropathy is supported by our understanding of monogenic lipodystrophy syndromes, which may be associated with neuropathy^29–31^, and indicates further superiority of pPS over PRS in uncovering rarer and clinically meaningful associations between etiology and disease outcomes.

Strengths of this study include its exploration of an underrepresented population with high burden of cardiometabolic disease and linkage of real-world electronic health record (EHR) and prescribing data, which provides a platform for real-world application and translation of clinically relevant findings, in addition to internal validation of novel findings around pharmacogenetic applications of pPSs. We also demonstrate the utility of pPSs derived by Smith et al.^16^ in a population not included in their pPS derivation. Weaknesses include the lack of external validation of results, which is limited, in part, by the paucity of non-European studies combining genetic data with health record data, particularly for South Asians^7^; in fact, some of the results shown, such as response to medication, have not even been shown in European cohorts due to rarity of required clinical and prescribing data, and case and complication numbers in the UK Biobank were inadequate for meaningful replication of results (Fig. 1). Another limitation is the fact that this is not an ancestry-specific pPS, which suggests that we might not be using the most optimal causal variants. However, this is likely to cause an underestimation of true genetic effects^32,33^. In common with all studies using real-world EHR data, there is a risk of misclassification of diabetes, miscoding and sampling bias toward individuals with chronic disease as well as the possibility of inaccuracies in dates of earliest timepoint of diagnosis and medication initiation, particularly for people who receive care outside of England. In using the presence or absence of clinical codes to define clinical phenotypes and progression to diabetes complications, our analyses are subject to the information bias that is a known feature of real-world health data analyses. This bias can arise from different sources—for example, the lack of comprehensive and systematic coding practice could mean an individual may have biochemical results consistent with nephropathy in their medical records but with no associated clinical code in their health record. Alternatively, the bias may arise from how a patient interacts with a health system—for example, an individual may have established retinopathy, but this may not have been detected or coded if they have not attended their routine eye screening. These biases are partially mitigated by the alignment of our clinical phenotypes to coding practice that is incentivized and structured in the UK National Health Service (NHS). Finally, it is possible that the Genes & Health study is subject to participant bias, as in the UK Biobank and other studies, with overrepresentation of wealthier, healthier and more educated individuals^34^. However, the community-based recruitment approach undertaken by Genes & Health has allowed it to recruit individuals from deprived areas of the UK broadly representative of the background population^19^. In general, there is a need for high-quality cohorts of South Asians (and other underrepresented ancestry groups) with deep clinical phenotyping and high-quality genetic and multi-omic data for replication and, more generally, to better understand ancestry-specific drivers of earlier onset and T2D. Investment in such resources has the potential to improve screening, diagnosis and management of T2D in these populations.

## Methods

### Cohort profile: discovery and replication cohorts

Genes & Health is a long-term, community-based study of British Pakistani and British Bangladeshi individuals aged 16 years and older living in the UK^19^. At recruitment, participants provide a saliva sample for genotyping, complete a short questionnaire on basic demographic information and consent to linkage for primary care, secondary care and national NHS EHRs. Since recruitment began in 2015, over 60,000 participants have been recruited, with linked genetic and EHR information available for 44,396 as of July 2023 (number of T2D cases = 9,771) and 50,556 as of February 2024. A participant flow diagram showing individuals included in analyses is shown in Fig. 1.

### Ethical approval

We conducted this research under an approved application to the Genes & Health Executive. The Genes & Health study is approved by the London South East NRES Committee of the Health Research Authority (14/LO/1240).

### Inclusion and exclusion criteria

We used no specific inclusion criteria. We excluded individuals with clinical codes consistent with type 1 diabetes, maturity-onset diabetes of the young (MODY) or causes of secondary diabetes, such as cystic fibrosis and pancreatectomy.

### Genetic data processing and curation

Genotyping was performed on Illumina Infinium Global Screening Array v3 with additional multi-disease variants. Quality control was performed following a standardized approach^33^. In brief, variants with call rates less than 0.99 and/or minor allele frequency (MAF) < 1% were excluded. We excluded individuals unlikely to have genetically inferred Pakistani or Bangladeshi ancestry. Imputation was performed using the TOPMed-r2 panel. We excluded SNPs with low imputation scores (INFO < 0.3) or MAF < 0.1%.

### Sex determination

We defined sex on the basis of XX (female) and XY (male) chromosomal presence in genotype data.

### EHR data processing and curation

We curated routine UK NHS EHR data from primary care (Systematized Nomenclature of Medicine (SNOMED) coded) and secondary care (International Classification of Diseases, Tenth Revision (ICD-10) coded) sources. Data were combined without mapping between coding formats. For each clinical code, we took the earliest ever measure recorded in a participant’s medical records, excluding erroneous code dates preceding the participant’s recorded date of birth.

### Exposures

#### pPS construction and ancestry correction

We used PLINK to calculate pPSs for 12 diabetes-associated genetically determined endotypes described by Smith et al.^16^, derived from high-throughput genetic clustering techniques in European (78%), African (19%) and East Asian (2.1%) ancestry individuals, using only SNPs above the authors’ specified inclusion threshold (cluster weight > 0.78), weighted by their cluster weights^16^. These comprise three endotypes related to glucose sensing, insulin secretion and insulin production (Beta Cell 1, Beta Cell 2 and Proinsulin, respectively); three clusters related to insulin resistance and unfavorable adiposity (Obesity, Lipodystrophy 1 and Lipodystrophy 2); and six clusters with unclear effects on insulin resistance and deficiency (Liver/Lipid, Alkaline Phosphatase (ALP) Negative, Hyper Insulin Secretion, Cholesterol, Sex Hormone-Binding Globulin Lipoprotein A (SHBG/LpA) and Bilirubin).

#### Regressing the effect of genetic PCs out of pPSs to allow direct comparison between British Pakistani and Bangladeshi individuals

PC analysis of genetic data shows distinct population structure for people of Bangladeshi and Pakistani ancestries^35^, and we observed differences in pPS distribution between these groups (Extended Data Fig. 1). Therefore, to maximize power and facilitate combined analyses of all individuals, we regressed out the effect of the first 10 genetic PCs from each pPS, using an approach described by Liu et al.^35^. In brief, we constructed residual PRSs and pPSs after regressing the first 10 genetic PCs out of each PRS and pPS separately in non-diabetic controls, after which no statistically significant differences between Pakistani and Bangladeshi distributions were observed (Extended Data Fig. 1). These residual PRSs and pPSs were used in all downstream analyses, except those exploring ancestry-specific pPS distributions (in which case the term ‘unmodified pPS’ is used). Although genetic PCs were subsequently included in sensitivity analyses downstream, these (as would be expected) had no statistically significant effect in any analysis employing residual PRSs or pPSs and were, therefore, not included or presented in principal analyses in this paper. When comparing distributions, we varied the applied test between ANOVA and Kruskal–Wallis depending on distribution normality.

#### T2D polygenic score

We additionally calculated scores for a global T2D PRS using a previously published score comprising 1,091,608 variants derived in European ancestry individuals^36^. We selected this PRS by calculating scores for all T2D PRSs published on the PGS Catalog^37^ and comparing performance, assessed as area under the receiver operating characteristic curve (estimated using the R package pROC) and beta, both estimated from multivariable logistic regression models describing score associations with incident T2D, adjusted for age, sex, ancestry and the first 10 genetic PCs. Score performance is summarized in Supplementary Table 12; the best-performing scores in Genes & Health were similar to those in European ancestry populations^38^. We corrected this score for genetically determined ancestry using the same process as that described above for the pPS.

#### pPS ‘extremes’

We defined pPS ‘extremes’ as scores in the top or bottom 10% of each residualized pPS distribution and ‘combined extremes’ as individuals with scores in the top or bottom 10% of multiple pPS distributions.

#### UK Biobank—cross-ancestry differences in genetic burden and viability for replication

We used the UK Biobank^39^ to compare the distribution of pPSs between individuals with T2D of European and South Asian ancestry; T2D was defined in line with established clinical codelists^40^. Differences in distribution of pPSs across ancestry groups were assessed using *t*-test for normally distributed pPSs and Wilcoxon signed-rank testing for all other pPSs. Data from individuals of European ancestry in the UK Biobank were included in the T2D GWAS, which defined genetic variants that were partitioned as part of the pPS discovery study^16^, and in a subset of phenotype GWAS used to define these pPSs. However, South Asian ancestry individuals were not included, likely due to small sample size. We provide a population flow chart showing the numbers of T2D cases split by ancestry and number of recorded complications to determine suitability for replication analyses (Fig. 1). Analyses in the UK Biobank were conducted under application IDs 44448 and 153692.

#### Medications

Diabetes-controlling medication classes were defined according to method of action: insulin secretagogues (sulfonylureas and meglitinide), incretin mimetics (GLP1 receptor agonists and DDP4 inhibitors), insulin sensitizers (pioglitazone and thiazolidinediones) and renal tubular glucose reabsorption modifiers (SGLT2 inhibitors) in addition to metformin and insulin. Initiation of medication was defined as the first instance of medication prescription in the EHR. For treatment response analyses (described in further detail in the ‘Outcomes’ subsection below), concurrently prescribed medications were defined as medications from another class prescribed within a conservative window of 6 months before or after initiation of the medication being treated as the exposure.

### Outcomes

#### Diabetes phenotypes and complications

Clinical phenotypes were defined on the basis of diagnostic codes present in the EHR. We used clinically curated ICD-10 and SNOMED codelists adapted from the AI-MULTIPLY resource^41^ using reproducible, consensus-derived methods to define all diabetes phenotypes and complications (Supplementary Table 13). Where appropriate, our curated codelists align to structured and incentivized clinical coding processes used in the UK NHS. Diabetes phenotypes included T2D, GDM and incident T2D after GDM. Diabetes-related complications were defined as microvascular (nephropathy, neuropathy and retinopathy) and macrovascular (coronary artery disease, cerebrovascular disease and peripheral vascular disease).

Diagnostic codes with unrealistic timestamps were removed (before or on date of birth or after the date of last data extraction). The earliest code date across primary and secondary care records was defined as the condition diagnosis date. Sex-specific codes applied to the wrong sex (for example, males with a diagnostic code of GDM) were removed.

T2D was defined as a clinical code of T2D in the EHR (Supplementary Table 13), entered after age 18 years, in the absence of excluder conditions (type 1 diabetes, MODY, cystic fibrosis, pancreatectomy, Cushing’s syndrome, Cushing’s disease and all documented cases of secondary diabetes). GDM was defined as a clinical code of GDM in the EHR of female participants; GDM codes occurring after a documented code of T2D, or any excluder conditions, were discounted. T2D after GDM was defined as incident T2D after GDM—that is, individuals for whom the earliest clinical code for GDM preceded the earliest clinical code for T2D. Individuals with GDM and T2D were not removed from T2D-specific analyses (Fig. 1).

Diabetes-related complications were defined as microvascular (nephropathy (*n* = 1,470), retinopathy (*n* = 4,764), neuropathy (*n* = 462)) and macrovascular (coronary artery disease (*n* = 2,606), cerebrovascular disease (*n* = 1,233) and peripheral vascular disease (*n* = 297)). Individuals with pre-existing complications at the time of T2D diagnosis were excluded from survival analyses—that is, only incident complications after T2D diagnosis were analyzed. Clinical codelists for these conditions were taken from the AI_MULTIPLY resource, a codelist tool developed using consensus methodology by local clinicians, including diabetologists and primary care doctors, designed to capture reasonable definitions of complications. For some complications that may be ambiguous— such as nephropathy, which lies on a spectrum of disease defined by estimated glomerular filtration rate and albuminuria, and retinopathy—the AI-MULTIPLY codelist sought to capture codes harmonizing with the UK Quality Outcomes Framework (QOF)—that is, the incentivized and structured approach to coding of these diabetes-related complications in routine healthcare in the UK^42^. Although these conditions may be described differently across different healthcare systems, nations and populations, the use of a robust and well-defined clinical codelist algorithm to define conditions allows for reproducibility of results and alignment with other populations using EHRs in the UK.

#### Age at diagnosis

Date of diagnosis for each outcome was defined as the earliest recorded date in either primary or secondary care above the age of 18 years.

#### Quantitative traits

Quantitative outcomes were, unless otherwise stated, defined as the measure taken closest to the date of T2D within 1 year (before and/or after) and included age, BMI, waist circumference, HbA1c, fasting and random blood glucose, low-density and high-density lipoprotein cholesterol, serum triglycerides, alkaline phosphatase (ALP) and alanine transaminase (ALT). Because diabetes-related traits may rapidly change after diagnosis and/or initiation of treatment, for each trait the value closest to the time of diagnosis was used. In addition to traits at diagnosis, we explored the number of medication classes (as defined above) that an individual was prescribed in 5 years and the change in HbA1c from time of diagnosis to 5 years (the HbA1c closest in time to 5 years from diagnosis date was taken, and only values between 4 years and 6 years after diagnosis were included in the analysis). Quantitative traits were processed as previously described, including exclusion of outliers lying 6 or more s.d. above or below the mean^43^.

#### Response to glucose-lowering treatment

For treatment response analyses, medication data were extracted from the primary care EHR. In line with pharmacogenomic studies^44^, treatment response was defined as the percentage change between the most recent HbA1c in 6 months before medication initiation and the lowest HbA1c in the 1 year after initiation, as a proportion of pre-medication HbA1c (that is, percent change from before initiation).

Diabetes-controlling medication classes were defined according to method of action: insulin secretagogues (sulfonylureas and meglitinide), insulin sensitizers (pioglitazone and thiazolidinediones) and renal tubular glucose reabsorption modifiers (SGLT2 inhibitors) in addition to metformin and insulin. Time to initiation of insulin was calculated as the time lag between the earliest T2D diagnostic code in the medical record and the earliest record of insulin prescription; insulin prescriptions with an earlier date than time of diabetes diagnosis were discarded.

### Statistical analyses

#### Descriptive analysis

We calculated mean values for quantitative traits at diagnosis and 5 years after diagnosis, stratified by ancestral group, and compared these using ANOVA.

#### Multivariable analysis

We described the association of each pPS (the exposure) with each diabetes phenotype outcome, using multivariable logistic regression models adjusted for age, sex and ancestry, to estimate the per-s.d. increase in odds of diabetes phenotype between diabetes phenotype cases and non-diabetic controls. We estimated the association of each pPS (the exposure) with diabetes-related traits at the time of diagnosis (the outcome). To allow comparison of effects of pPS between quantitative traits at the time of diagnosis, each quantitative trait was scaled to a normal distribution, and the beta per s.d. of pPS was presented for each trait, estimated from multivariable logistic regression models adjusted for age, sex and ancestry. Multivariable linear regression was used to estimate the effect of pPS on age of T2D diagnosis, adjusted for ancestry and sex; partial R^2^ was calculated with the R package ‘partialR2’. We assumed a priori that associations may differ between sexes and ancestry groups; because of this, sex-stratified and ancestry-stratified analyses were also performed. For pharmacogenomic analysis of treatment response, association between each pPS (the exposure) and HbA1c change in response to medication (the outcome) was estimated from multivariable logistic regression models adjusted for age, sex and ancestry as well as concurrently prescribed anti-diabetic medication from all other classes within 6 months before or after initiation of each. We meta-analyzed treatment response analyses from the discovery and replication samples using fixed effects models with the R package ‘metafor’.

#### Survival analysis

We constructed survival models starting from each individual’s date of diagnosis, running until last data extraction, for two outcome categories: initiation of insulin and progression to diabetes-related complications. We explored the association of each pPS with each complication outcome using Cox proportional hazard models adjusted for age, sex and ancestry. We calculated Schoenfeld residuals for each model to check assumptions of proportionality.

#### Bonferroni correction for multiple testing

Analyses reported in this paper tested associations of multiple polygenic scores with multiple outcomes. Where appropriate, we present analysis-specific Bonferroni-corrected *P* values, calculated as *P* = 0.05 / (number of associations tested in analysis).

#### Software and statistical computing

Genotype curation and pPS calculation were performed using PLINK version 2.0 (ref. ^45^). Statistical analyses were performed using R version 4.2.3.

#### Reporting

We report this study following the STREGA^46^ and SAGER^47^ guidelines.

### Reporting summary

Further information on research design is available in the Nature Portfolio Reporting Summary linked to this article.

## Online content

Any methods, additional references, Nature Portfolio reporting summaries, source data, extended data, supplementary information, acknowledgements, peer review information; details of author contributions and competing interests; and statements of data and code availability are available at 10.1038/s41591-024-03317-8.

## Supplementary information

Supplementary Information Contents, Supplementary Results, STREGA Checklist, SAGER Checklist and References

Reporting Summary

Supplemental Table Supplementary Tables 1–15

## Data Availability

Genes & Health: Individual-level participant data are available to researchers and industry partners worldwide via application to and review by the Genes & Health Executive (https://www.genesandhealth.org/); applications are reviewed monthly. Approved researchers have access to individual-level data in the Genes & Health Trusted Research Environment (TRE) and can request the data files used in this study from the corresponding author(s). All data exports from the Genes & Health TRE are reviewed to prevent release of identifiable individual-level data. Summary data may be exported for cross-cohort meta-analysis or replication and for publication, subject to review. UK Biobank: All individual-level data are available to bona fide researchers from the UK Biobank upon application (https://www.ukbiobank.ac.uk/). All summary statistics were previously published in supplementary materials.

## Code availability

All code used for reported analyses can be made available upon reasonable request to corresponding author(s) with an estimated timeframe of 1 month to respond. We did not develop any new code or analysis packages; where appropriate, the packages and processes used are referenced. We used pPSs developed by Smith et al.^16^, and no de novo packages were required or created for analyses.
