# Supplementary material for: Genetic basis of early onset and progression of type 2 diabetes in South Asians
Source: Nat Med. 2024 Nov 26;31(1):323–31. doi: 10.1038/s41591-024-03317-8 (PMC11750703; doi:10.1038/s41591-024-03317-8)
Supplement: Supplementary file 1 — Contents, Supplementary Results, STREGA Checklist, SAGER Checklist and References [file 41591_2024_3317_MOESM1_ESM.pdf]

nature medicine

Supplementary information https://doi.org/10.1038/s41591-024-03317-8

Genetic basis of early onset and progression of type 2 diabetes in South Asians

In the format provided by the authors and unedited

Supplementary information

Table of Contents:

1) Supplementary results
2) STREGA Checklist
3) SAGER Checklist
4) References

1) Supplementary results

Association of partitioned polygenic scores and quantitative traits at time of diagnosis

We observed numerous expected associations between single pPS and traits at time of diagnosis (**Fig S3, Table S2)**,including associations between beta cell related pPSs (Beta Cell 1, Beta Cell 2, Proinsulin), and lower BMI, and greater HbA1c, fasting and random blood glucose at diagnosis; these findings were in keeping with associations previously reported in multi-ancestry analyses principally comprising European, African and East Asian ancestry individuals[1]. Lipodystrophy 1 was associated with lower BMI and waist circumference and raised ALT at time of diagnosis. Both lipodystrophy-related pPSs were associated with lower HDL and raised triglycerides, whereas the Cholesterol pPS was associated with raised HDL and lower triglycerides. The Obesity pPS was associated with greater BMI and waist circumferences.

Several pPS demonstrated reasonably strong correlations with one another (**Fig S4, Table S3)**, with strongest associations observed between pPS with similar proposed mechanisms of action, eg between Lipodystrophy 1 and Lipodystrophy 2 (Pearson R^2 = 0.50), and for Beta Cell 1 and Beta Cell 2 (R^2 = 0.46). Weaker correlations were observed between pPS acting via distinct pathophysiological pathways, e.g. Beta Cell 1 and Lipodystrophy 1 (R^2 = 0.35), and Beta Cell 1 and Obesity (R^2 = 0.31).

<u>Genetic Risk Extremes and Progression To Complications: pPS but not the T2D PRS Are Associated With Lean and Early Onset Diabetes</u>

While individuals in the top decile of the T2D PRS distribution (n = 1365) were diagnosed at a younger age than those in the bottom decile of the distribution (n = 508), the mean age of diagnosis of those at high T2D genetic risk (43.5 years) was higher than those at combined high genetic risk of Lipodystrophy 1 and Beta Cell 2 (42.5 years) (**Table S8, Fig 5A)**. We observed no difference in BMI distribution between the top and bottom deciles of the T2D PRS distribution (**Fig 5B).** The T2D PRS was most strongly associated with progression to diabetic nephropathy, and was associated with coronary artery disease (although not passing Bonferroni correction), but not progression to diabetic neuropathy (**Fig 5C).**
Among all 9771 individuals with T2D included in analyses, continuous pPS scores (as opposed to genetic risk extremes) were also associated with progression to complications **(Table S12).** After adjustment for multiple testing, both the Beta Cell 2 pPS and T2D PRS were associated with progression to nephropathy (HR = 1.13 and 1.19, p = 1e-5 and 1e-6, respectively). Several other nominally significant associations did not pass Bonferroni correction, including associations between cardiovascular disease and T2D PRS, Beta Cell 2 and Obesity; between nephropathy and Obesity and Beta Cell 1; and between SHBG/LpA and neuropathy (**Table S11).**

Association of number of Single Nucleotide Polymorphisms (SNPs) in each pPS with reported results

In response to peer review, we explored whether the pPS associations with diabetes-related binary outcomes (T2D, GDM, and T2D after GDM (results shown in **Fig 1)** and T2D age of diagnosis (results shown in **Fig 2)** were associated with the number of SNPs present in each pPS, ie, are stronger associations driven simply by larger numbers of SNPs within each pPS. In addition to total number of SNPs, we also considered the total sum of weights assigned to each SNP in pPS score calculations (total SNP weight for each pPS), and the mean SNP weight (total SNP weight / number of SNPs) (**Table S14)**.

To explore whether these three metrics may be associated with strength of our reported associations, we took the p values obtained in **Fig 2** and **Fig 3** (associations of pPS with T2D/GDM/T2D after GDM, and of pPS with T2D age of diagnosis). We then regressed these p values on each of these metrics in turn.

We observed no association between total number of SNPs and strength of reported association for these analyses, nor did we observe an association between total SNP weight and strength of association (**Table S15)**. Mean SNP weight was associated with p value but this was a negative association. Our conclusion was that this evidence does not support the hypothesis that number of SNPs (nor their weights) is a clear feature driving the associations of pPS with T2D, GDM, or age of diagnosis.

2) Reporting checklist for genetic association study.

Based on the STREGA guidelines.

Instructions to authors

Complete this checklist by entering the page numbers from your manuscript where readers will find each of the items listed below.

Your article may not currently address all the items on the checklist. Please modify your text to include the missing information. If you are certain that an item does not apply, please write "n/a" and provide a short explanation.

Upload your completed checklist as an extra file when you submit to a journal.

In your methods section, say that you used the STREGAreporting guidelines, and cite them as:

Little J, Higgins JP, Ioannidis JP, Moher D, Gagnon F, von Elm E, Khoury MJ, Cohen B, Davey-Smith G, Grimshaw J, Scheet P, Gwinn M, Williamson RE, Zou GY, Hutchings K, Johnson CY, Tait V, Wiens M, Golding J, van Duijn C, McLaughlin J, Paterson A, Wells G, Fortier I, Freedman M, Zecevic M, King R, Infante-Rivard C, Stewart A, Birkett N; STrengthening the REporting of Genetic Association Studies. STrengthening the REporting of Genetic Association Studies (STREGA): An Extension of the STROBE Statement.

		Reporting Item	Page Number
**Title and abstract**			
Title	#1a	Indicate the study's design with a commonly used term in the title or the abstract	1
Abstract	#1b	Provide in the abstract an informative and balanced summary of what was done and what was found	1
**Background/rationale**			
	#2	Explain the scientific background and rationale for the investigation being reported	2
**Objectives**			
	#3	State specific objectives, including any prespecified hypotheses. State if the study is the first report of a genetic association, a replication effort, or both.	2-3
**Study design**			
	#4	Present key elements of study design early in the paper	3
**Setting**			
	#5	Describe the setting, locations, and relevant dates, including periods of recruitment, exposure, follow-up, and data collection	3
**Eligibility criteria**			
	#6a	Cohort study – Give the eligibility criteria, and the sources and methods of selection of participants. Describe methods of follow-up. Case-control study – Give the eligibility criteria, and the sources and methods of case ascertainment and control selection. Give the rationale for the choice of cases and controls. Cross-sectional study – Give the eligibility criteria, and the sources and methods of selection of participants. Give information on the criteria and methods for selection of subsets of participants from a larger study, when relevant.	12

	#6b	Cohort study – For matched studies, give matching criteria and number of exposed and unexposed. Case-control study – For matched studies, give matching criteria and the number of controls per case.	12,13
**Variables**			
	#7a	Clearly define all outcomes, exposures, predictors, potential confounders, and effect modifiers. Give diagnostic criteria, if applicable	13-16
	#7b	Clearly define genetic exposures (genetic variants) using a widely-used nomenclature system. Identify variables likely to be associated with population stratification (confounding by ethnic origin).	13-17
**Data sources/measurement**			
	#8a	For each variable of interest give sources of data and details of methods of assessment (measurement). Describe comparability of assessment methods if there is more than one group. Give information separately for for exposed and unexposed groups if applicable.	13-16
	#8b	Describe laboratory methods, including source and storage of DNA, genotyping methods and platforms (including the allele calling algorithm used, and its version), error rates and call rates. State the laboratory / centre where genotyping was done. Describe comparability of laboratory methods if there is more than one group. Specify whether genotypes were assigned using all of the data from the study simultaneously or in smaller batches.	12
**Bias**			

	#9a	Describe any efforts to address potential sources of bias	11,14
	#9b	Describe any efforts to address potential sources of bias	11,14
**Study size**			
	#10	Explain how the study size was arrived at	4,12
**Quantitative variables**			
	#11	Explain how quantitative variables were handled in the analyses. If applicable, describe which groupings were chosen, and why. If applicable, describe how effects of treatment were dealt with.	15
**Statistical methods**			
	#12a	Describe all statistical methods, including those used to control for confounding. State software version used and options (or settings) chosen.	16-17
	#12b	Describe any methods used to examine subgroups and interactions	12-17
	#12c	Explain how missing data were addressed	13
	#12d	If applicable, explain how loss to follow-up was addressed	NA
	#12e	Describe any sensitivity analyses	12-17, Extended results
	#12f	State whether Hardy-Weinberg equilibrium was considered and, if so, how.	NA
	#12g	Describe any methods used for inferring genotypes or haplotypes	12
	#12h	Describe any methods used to assess or address population stratification.	13
	#12i	Describe any methods used to address multiple comparisons or to control risk of false positive findings.	17
	#12j	Describe any methods used to address and correct for relatedness among subjects	NA
**Participants**			

	#13a	Report numbers of individuals at each stage of study—eg numbers potentially eligible, examined for eligibility, confirmed eligible, included in the study, completing follow-up, and analysed. Give information separately for for exposed and unexposed groups if applicable. Report numbers of individuals in whom genotyping was attempted and numbers of individuals in whom genotyping was successful.	5, Fig S11
	#13b	Give reasons for non-participation at each stage	5, Fig S11
	#13c	Consider use of a flow diagram	Fig S11
**Descriptive data**			
	#14a	Give characteristics of study participants (eg demographic, clinical, social) and information on exposures and potential confounders. Give information separately for exposed and unexposed groups if applicable. Consider giving information by genotype	Table 1
	#14b	Indicate number of participants with missing data for each variable of interest	Supplementary methods
	#14c	Cohort study – Summarize follow-up time, e.g. average and total amount.	Supplementary methods
**Outcome data**			
	#15	Cohort study Report numbers of outcome events or summary measures over time.Give information separately for exposed and unexposed groups if applicable. Report outcomes (phenotypes) for each genotype category over time Case-control study – Report numbers in each exposure category, or summary measures of exposure.Give information separately for cases and controls . Report numbers in each genotype category. Cross-	Supplementary methods

		sectional study – Report numbers of outcome events or summary measures. Give information separately for exposed and unexposed groups if applicable. Report outcomes (phenotypes) for each genotype category	
**Main results**			
	#16a	Give unadjusted estimates and, if applicable, confounder-adjusted estimates and their precision (eg, 95% confidence interval). Make clear which confounders were adjusted for and why they were included	Abstract, 3-8
	#16b	Report category boundaries when continuous variables were categorized	Abstract, 3-8
	#16c	If relevant, consider translating estimates of relative risk into absolute risk for a meaningful time period	NA
	#16d	Report results of any adjustments for multiple comparisons	NA
**Other analyses**			
	#17a	Report other analyses done—e.g., analyses of subgroups and interactions, and sensitivity analyses	3-8, extended results
	#17b	Report other analyses done—e.g., analyses of subgroups and interactions, and sensitivity analyses	3-8, extended results
	#17c	Report other analyses done—e.g., analyses of subgroups and interactions, and sensitivity analyses	3-8, extended results
**Key results**			
	#18	Summarise key results with reference to study objectives	13
**Limitations**			
	#19	Discuss limitations of the study, taking into account sources of potential bias or imprecision. Discuss both direction and magnitude of any potential bias.	11-12

**Interpretation**			
	#20	Give a cautious overall interpretation considering objectives, limitations, multiplicity of analyses, results from similar studies, and other relevant evidence.	8-12
**Generalisability**			
	#21	Discuss the generalisability (external validity) of the study results	11-12
**Funding**			
	#22	Give the source of funding and the role of the funders for the present study and, if applicable, for the original study on which the present article is based	Funding statement

None The STREGA checklist is distributed under the terms of the Creative Commons Attribution License CC-BY. This checklist can be completed online using https://www.goodreports.org/, a tool made by the EQUATOR Network in collaboration with Penelope.ai

3) SAGER Checklist

ease publications

Sex and Gender Equity in Research Guidelines Checklist

EASE Gender Policy Committee

The Sex and Gender Equity in Research (SAGER) Guidelines[1] are a comprehensive procedure for reporting of sex and gender information in study design, data analysis, results and interpretations of findings. They are primarily designed to guide authors in preparing their manuscripts but they are also useful for editors to integrate assessment of sex and gender in all manuscripts as an integral part of the editorial process.

The two checklists in this document are developed from the guidelines presented in the original SAGER article, with additional requirements identified by some *Lancet* journals.[2] They present a convenient list of items to check off when writing, reviewing or editing manuscripts.

One checklist is intended for studies including human participants and one for studies that do not include human participants, such as those using animals and cells.

This checklist was originally published as an article in the EASE journal, European Science Editing[2].

References

1. Heidari, S., Babor, T.F., De Castro, P., Tort S., Curno M. (2016) Sex and Gender Equity in Research: Rationale for the SAGER Guidelines and Recommended Use. Research Integrity and Peer Review, **1:** 2. https://doi.org/10.1186/s41073016-0007-6

2. Van Epps H, Astudillo O, Del Pozo Martin Y, Marsh J (2022) The Sex and Gender Equity in Research (SAGER) Guidelines: Implementation and Checklist Development. European Science Editing, 48: E86910. https://doi.org/10.3897/ese.2022.e86910

Authors

This checklist was created by Heather Van Epps*, Olaya Astudillo*, Yaiza Del Pozo Martín, and Joan Marsh, of the EASE Gender Policy Committee.

*Joint first authorship

Affiliations:

The Lancet Rheumatology (H Van Epps)

The Lancet (O Astudillo)

The Lancet Haematology (Y Del Pozo Martín)

The Lancet Psychiatry (J Marsh)

About the EASE Gender Policy Committee

Established in 2012, the EASE Gender Policy Committee works to advance gender- and sex-sensitive reporting and communication in science. The goal is not only better science, whether in the life, natural or social sciences, but also enhanced evidence-based practices, interventions and opportunities, for everyone. The Committee consists of experts with diverse backgrounds, linguistic traditions and professional experience, sharing enthusiasm to advance sex and gender equity for responsible research and innovation.

Read more about the committee on their EASE Community web pages: https://ease.org.uk/communities/gender-policy-committee/about-thiscommittee/

Table 1. SAGER guidelines checklist Studies with human participants

**Section / topic**	**Item number**	**Checklist item**	**Reported on page number**
**General**			

	1	The terms sex/gender used appropriately	Throughout
**Title**			
	2	Title specifies the sex/gender of participants if only one included	N/A
**Abstract**			
	3a	Abstract specifies the sex/gender of participants if only one included	N/A
	3b	Study population described with sex/gender breakdown*	Table 1
**Introduction**			
	4a	If relevant, previous studies that show presence or lack of sex/gender differences or similarities are cited	**8-12**
	4b	Mention of whether sex/gender might be an important variant and if differences might be expected	**4-5**
	4c	The demographics of the study population with regard to sex/gender (eg, disease prevalence among male/female study participants) are outlined*	**Table 1**
**Methods**			
	5a	Method of definition of sex/gender (eg, self-report, genetic testing)	**13 (sex defined via genetic testing)**
	5b	Description of how sex/gender was considered in the design, whether authors ensured adequate representation of male and female study participants, justification of the reasons for any exclusion of male or female participants, or explanation if not considered. Justification of other sex/gender-specific interventions of study designs (eg, mandating contraception for women).* Explicit reporting of the scientific rationale for	**Pregnancy / gestational diabetes mellitus explicitly included as an outcome; Pages 14-16**
		contraception requirements and exclusions for pregnancy and lactation should be required*	No exclusions
**Results**			
	6a	Study population description with complete gender/sex breakdown for all categories considered*	**Table 1**

	6b	Where appropriate, data presented disaggregated by sex/gender, and sex/gender differences and similarities are described	**Table 1, Fig S7**
	6c	Sex- and gender-based analyses reported regardless of outcome<br>(in main paper if pre-specified; otherwise in appendix)*	**Table 1, Fig S7**
	6d	For clinical trials, adverse event data disaggregated by sex/gender<br>(in main paper if pre-specified; otherwise in appendix)*	**N/A**
	6e	Patient-reported outcome data disaggregated by sex/gender (in main paper if pre-specified; otherwise in appendix)*	**N/A**
	6f	For epidemiological studies, the effects of other exposures on health problems examined for all genders and analysed critically from a gender perspective	**Table 1, Fig S7**
	6g	Table 1 includes separate rows for male sex/gender, female sex/gender and other categories if collected*	**Yes**
**Discussion**			
	7a	Potential implications of sex/gender on the study results and analyses, including the extent to which the findings can be generalized to all sexes/genders in a population	**8-12**
	7b	If a sex/gender analysis not done, a rationale is given and implications of the lack of such analysis on the interpretation of the results are discussed	**N/A**
Adapted from SAGER guidelines. Sex and Gender Equity in Research: rationale for the SAGER guidelines and recommended use. Research Integrity and Peer Review 1, Article number: 2 (2016) https://researchintegrityjournal.biomedcentral.com/articles/10.1186/s41073-016-0007-6.<br>* These points extend beyond the original SAGER table			

Table 2. SAGER guidelines checklist

Other studies (applied sciences, biological sciences)

Section / topic	Item number	Checklist item	Reported on page number
**General**			

	1	The terms sex/gender used appropriately	
**Title**			
	2a	Title specifies the sex of animals or any cells, tissues, and other material derived from these	
	2b	In applied sciences (technology, engineering, etc.), the title indicates if the study model was based on one sex/gender or the application was considered for the use of one specific sex/gender	
**Abstract**			
	3a	Abstract specifies sex of animals or any cells, tissues, and other material derived from these	
	3b	In applied sciences (technology, engineering, etc.), the abstract indicates if the study model was based on one sex/gender or the application was considered for the use of one specific sex/gender	
**Introduction**			
	4a	If relevant, previous studies that show presence or lack of sex or gender differences or similarities are cited	
	4b	Mention of whether sex/gender might be an important variant and if differences might be expected	
**Methods**			
	5a	In cell biological, molecular biological, or biochemical experiments, the origin and sex chromosome constitutions of cells or tissue cultures are stated. If unknown, the reasons are stated	
	5b	For studies testing devices or technology, explanation of whether the product will be applied or used by all genders and if it has been tested with a user's gender in mind	
	5c	If relevant, description of how sex/gender was considered in the design	

	5d	For in-vivo and in-vitro studies using primary cultures of cells, or cell lines from humans or animals, or ex-vivo studies with tissues from humans or animals, the sex of the subjects or source donors is stated (except for immortalized cell lines, which are highly transformed)	
**Results**			
	6	For studies using animal models, present a sex breakdown of the animals*	
**Discussion**			
	7	If relevant, potential implications of sex/gender on the study results and analyses, including the extent to which the findings can be generalized to all sexes/genders in a population	
Adapted from SAGER guidelines. Sex and gender equity in research: rationale for the SAGER guidelines and recommended use. Research Integrity and Peer Review 1, Article number: 2 (2016) https://researchintegrityjournal.biomedcentral.com/articles/10.1186/s41073-016-0007-6.<br>*These points extend beyond the original SAGER table.			

4) REFERENCES

1. Smith, K. *et al.* Multi-ancestry polygenic mechanisms of type 2 diabetes. *Nat. Med.* (2024) doi:10.1038/s41591-024-02865-3.
2. Bycroft, C. *et al.* The UK Biobank resource with deep phenotyping and genomic data. *Nature* **562**, 203–209 (2018).
3. Gardner, E. J. *et al.* Damaging missense variants in IGF1R implicate a role for IGF-1 resistance in the etiology of type 2 diabetes. *Cell Genom* **2**, None (2022).
