# Supplementary material for: Genetic basis of early onset and progression of type 2 diabetes in South Asians
Source: Nat Med. 2024 Nov 26;31(1):323–31. doi: 10.1038/s41591-024-03317-8 (PMC11750703; doi:10.1038/s41591-024-03317-8)
Supplement: Supplementary file 2 — Reporting Summary [file 41591_2024_3317_MOESM2_ESM.pdf]

nature portfolio

Corresponding author(s): Moneeza Siddiqui

Last updated by author(s): Aug 25, 2024

Reporting Summary

Nature Portfolio wishes to improve the reproducibility of the work that we publish. This form provides structure for consistency and transparency in reporting. For further information on Nature Portfolio policies, see our Editorial Policies and the Editorial Policy Checklist.

Statistics

For all statistical analyses, confirm that the following items are present in the figure legend, table legend, main text, or Methods section.

n/a	Confirmed	
☐	☒	The exact sample size (*n*) for each experimental group/condition, given as a discrete number and unit of measurement
☐	☒	A statement on whether measurements were taken from distinct samples or whether the same sample was measured repeatedly
☐	☒	The statistical test(s) used AND whether they are one- or two-sided<br>*Only common tests should be described solely by name; describe more complex techniques in the Methods section.*
☐	☒	A description of all covariates tested
☐	☒	A description of any assumptions or corrections, such as tests of normality and adjustment for multiple comparisons
☐	☒	A full description of the statistical parameters including central tendency (e.g. means) or other basic estimates (e.g. regression coefficient) AND variation (e.g. standard deviation) or associated estimates of uncertainty (e.g. confidence intervals)
☐	☒	For null hypothesis testing, the test statistic (e.g. *F*, *t*, *r*) with confidence intervals, effect sizes, degrees of freedom and *P* value noted<br>*Give P values as exact values whenever suitable.*
☒	☐	For Bayesian analysis, information on the choice of priors and Markov chain Monte Carlo settings
☒	☐	For hierarchical and complex designs, identification of the appropriate level for tests and full reporting of outcomes
☐	☒	Estimates of effect sizes (e.g. Cohen's *d*, Pearson's *r*), indicating how they were calculated

Our web collection on statistics for biologists contains articles on many of the points above.

Software and code

Policy information about availability of computer code

Data collection	We used pre-collected data from the Genes & Health study and UK Biobank. We did not use any software or code for data collection. We provide a praisie for these cohort studies in the methods section.
Data analysis	Genotype curation and partitioned polygenic risk score calculation was performed using Plink v2.028. Statistical analyses were performed using R V4.2.3, including packages “PartialR2(v1.9-7)”, “Survival(v3.7-0)”, “fmsb(v0.7.6)”, "pROC(v1.18.5)", and “metafor(v4.6-0)”. We did not create any custom programmes or packages for analysis. We did not develop any custom software.

For manuscripts utilizing custom algorithms or software that are central to the research but not yet described in published literature, software must be made available to editors and reviewers. We strongly encourage code deposition in a community repository (e.g. GitHub). See the Nature Portfolio guidelines for submitting code & software for further information.

Data

Policy information about availability of data

All manuscripts must include a data availability statement. This statement should provide the following information, where applicable:

- Accession codes, unique identifiers, or web links for publicly available datasets
- A description of any restrictions on data availability
- For clinical datasets or third party data, please ensure that the statement adheres to our policy

Genes & Health: Individual level participant data is available to researchers and industry partners worldwide via application to and review by the Genes & Health

Executive (https://www.genesandhealth.org/); applications are reviewed monthly. Approved researchers have access to individual level data in the Genes & Health Trusted Research Environment (TRE), and can request the data files used in this study from the corresponding author(s). All data exports from the Genes & Health TRE are reviewed to prevent release of identifiable individual level data. Summary data may be exported for cross-cohort meta-analysis or replication and for publication, subject to review. UK Biobank: All individual-level data is available to bona fide researchers from the UK Biobank upon application (https://www.ukbiobank.ac.uk/).
All summary statistics have been previously published in supplementary materials.

Research involving human participants, their data, or biological material

Policy information about studies with human participants or human data. See also policy information about sex, gender (identity/presentation), and sexual orientation and race, ethnicity and racism.

Reporting on sex and gender	We use the term sex throughout this manuscript; this was determined based on genetic presence or absence of X and Y chromosomes. We have completed the SAGER checklist which is available alongside this reporting summary and the main manuscript.
Reporting on race, ethnicity, or other socially relevant groupings	We use the term ancestry throughout this manuscript, and define this as genetically-inferred ancestry on the basis of principal component analysis.
Population characteristics	We present summary demographic information for included participants in table 1, and this is described in greater detail in the cohort paper referenced at the start of the methods. In brief, Genes & Health contains 51,170 British Pakistani and Bangladeshi individuals (63.1% British Bangladeshi, 54.8% female, mean age at recruitment 40.3 years (SD 13.0 years). These individuals have been genotyped using the Illumina Infinuim Global ScreeningGSA Array V3, imputed to TOPMED -R2. For UKBiobank, we include 7,491 South Asian individuals (22% Pakistani, 4% Bangladeshi, remainder British Indian), mean age at recruitment 56.5 years (SD 8.09 years), imputed to TOPMED-R2.
Recruitment	Genes & Health is a community-based study recruiting participants from British Pakistani and Bangladeshi backgrounds in the United Kingdom. Volunteers are recruited from a variety of sites including primary and secondary care facilities, community settings such as mosques and community centres, and community outreach events, in great part driven by a dedicated community engagement team who are part of the local British Pakistani and Bangladeshi communities.
Ethics oversight	We conducted this research under an approved application to the Genes & Health Executive. The Genes & Health study is approved by the London South East NRES Committee of the Health Research Authority (14/LO/1240).

Note that full information on the approval of the study protocol must also be provided in the manuscript.

Field-specific reporting

Please select the one below that is the best fit for your research. If you are not sure, read the appropriate sections before making your selection.

☒ Life sciences ☐ Behavioural & social sciences ☐ Ecological, evolutionary & environmental sciences

For a reference copy of the document with all sections, see nature.com/documents/nr-reporting-summary-flat.pdf

Life sciences study design

All studies must disclose on these points even when the disclosure is negative.

Sample size	We used the maximum available sample size in Genes & Health. At the time of preliminary analyses 44,189 individuals were present in data-sets with linked electronic health record and genotyping data. During the analysis process, an additional data-freeze of 7383 newly genotyped individuals, of whom 1907 had T2D, was released. We used this additional sample as a "replication" sample to replicate key findings.
Data exclusions	We excluded electronic healthcare record codes with unrealistic timepoints (eg prior to date of birth); and excluded individuals with clinical codes consistent with type 1 diabetes, MODY diabetes, or causes of secondary diabetes (such as cystic fibrosis / pancreatectomy).
Replication	We repeated key analyses in a separate sample of 7883 individuals not present in our initial analyses, but from the same data-set (Genes & Health); these individuals were added to the data-set as part of an updated data-freeze. We included all analyses with attempted replication in the final manuscript, and meta-analysed results between initial "discovery" and "replication" analyses. 4 of the 5 findings observed in discovery datasets replicated.
Randomization	Randomization was not applicable to this study as we were not assigning participants to groups for intervention or analysis; rather, we performed retrospective analyses on existing data.
Blinding	Blinding was not applicable to this study as we were not assigning participants to groups for intervention or analyses. This was a retrospective cohort study design.

Reporting for specific materials, systems and methods

We require information from authors about some types of materials, experimental systems and methods used in many studies. Here, indicate whether each material, system or method listed is relevant to your study. If you are not sure if a list item applies to your research, read the appropriate section before selecting a response.

Materials & experimental systems

n/a	Involved in the study
☒	☐ Antibodies
☒	☐ Eukaryotic cell lines
☒	☐ Palaeontology and archaeology
☒	☐ Animals and other organisms
☒	☐ Clinical data
☒	☐ Dual use research of concern
☒	☐ Plants

Methods

n/a	Involved in the study
☒	☐ ChIP-seq
☒	☐ Flow cytometry
☒	☐ MRI-based neuroimaging

Plants

Seed stocks	NA
Novel plant genotypes	NA
Authentication	NA
